# Id4, a New Candidate Gene for Senile Osteoporosis, Acts as a Molecular Switch Promoting Osteoblast Differentiation

**DOI:** 10.1371/journal.pgen.1001019

**Published:** 2010-07-08

**Authors:** Yoshimi Tokuzawa, Ken Yagi, Yzumi Yamashita, Yutaka Nakachi, Itoshi Nikaido, Hidemasa Bono, Yuichi Ninomiya, Yukiko Kanesaki-Yatsuka, Masumi Akita, Hiromi Motegi, Shigeharu Wakana, Tetsuo Noda, Fred Sablitzky, Shigeki Arai, Riki Kurokawa, Toru Fukuda, Takenobu Katagiri, Christian Schönbach, Tatsuo Suda, Yosuke Mizuno, Yasushi Okazaki

**Affiliations:** 1 Division of Functional Genomics and Systems Medicine, Research Center for Genomic Medicine, Saitama Medical University, Hidaka, Saitama, Japan; 2 Division of Morphological Science, Biomedical Research Center, Saitama Medical University, Iruma-gun, Saitama, Japan; 3 RIKEN BioResource Center, Tsukuba, Ibaraki, Japan; 4 The Cancer Institute of the Japanese Foundation for Cancer Research, Koto-ward, Tokyo, Japan; 5 Developmental Genetics and Gene Control, Institute of Genetics, University of Nottingham, Queen's Medical Center, Nottingham, United Kingdom; 6 Division of Gene Structure and Function, Research Center for Genomic Medicine, Saitama Medical University, Hidaka, Saitama, Japan; 7 Division of Pathophysiology, Research Center for Genomic Medicine, Saitama Medical University, Hidaka, Saitama, Japan; 8 Division of Genomics and Genetics, Nanyang Technological University School of Biological Sciences, Singapore, Singapore; 9 Department of Bioscience and Bioinformatics, Kyushu Institute of Technology, Iizuka, Fukuoka, Japan; Stanford University School of Medicine, United States of America

## Abstract

Excessive accumulation of bone marrow adipocytes observed in senile osteoporosis or age-related osteopenia is caused by the unbalanced differentiation of MSCs into bone marrow adipocytes or osteoblasts. Several transcription factors are known to regulate the balance between adipocyte and osteoblast differentiation. However, the molecular mechanisms that regulate the balance between adipocyte and osteoblast differentiation in the bone marrow have yet to be elucidated. To identify candidate genes associated with senile osteoporosis, we performed genome-wide expression analyses of differentiating osteoblasts and adipocytes. Among transcription factors that were enriched in the early phase of differentiation, Id4 was identified as a key molecule affecting the differentiation of both cell types. Experiments using bone marrow-derived stromal cell line ST2 and *Id4*-deficient mice showed that lack of *Id4* drastically reduces osteoblast differentiation and drives differentiation toward adipocytes. On the other hand knockdown of *Id4* in adipogenic-induced ST2 cells increased the expression of *Pparγ2*, a master regulator of adipocyte differentiation. Similar results were observed in bone marrow cells of femur and tibia of *Id4*-deficient mice. However the effect of *Id4* on *Pparγ2* and adipocyte differentiation is unlikely to be of direct nature. The mechanism of Id4 promoting osteoblast differentiation is associated with the Id4-mediated release of Hes1 from Hes1-Hey2 complexes. Hes1 increases the stability and transcriptional activity of Runx2, a key molecule of osteoblast differentiation, which results in an enhanced osteoblast-specific gene expression. The new role of Id4 in promoting osteoblast differentiation renders it a target for preventing the onset of senile osteoporosis.

## Author Summary

Increased bone marrow adiposity is observed in the bone marrow of senile osteoporosis patients. This is caused by unbalanced differentiation of mesenchymal stem cells (MSCs) into osteoblast or adipocyte. Previous reports have indicated that several transcription factors play important roles in determining the direction of MSCs differentiation into osteoblast or adipocyte. So far, little is known about the overall dynamics and regulation of transcription factor expression changes leading to the imbalance of osteoblast and adipocyte differentiation inside the bone marrow. We have performed genome-wide gene expression analyses during the differentiation of MSCs into osteoblast or adipocyte. We identified basic helix-loop-helix transcription factor family member Id4 as a leading candidate controlling the differentiation toward adipocyte or osteoblast. Suppression of Id4 expression in MSCs repressed osteoblast differentiation and increased adipocyte differentiation. In contrast, overexpression of Id4 in MSCs promoted osteoblast differentiation and attenuated adipocyte differentiation. Moreover, *Id4*-mutant mice showed abnormal accumulation of lipid droplets in bone marrow and impaired bone formation activity. In summary, we have demonstrated a molecular function of Id4 in osteoblast differentiation. The findings revealed that Id4 is a molecular switch enhancing osteoblast differentiation at the expense of adipocyte differentiation.

## Introduction

Senile osteoporosis or age-related osteopenia is accompanied by increased bone marrow tissue adiposity [1]. Bone marrow adipocytes and osteoblasts are thought to originate from common mesenchymal stem cells (MSCs). Therefore, it has been suggested that the excessive accumulation of marrow adipocytes following bone loss is caused by unbalanced differentiation of MSCs into marrow adipocytes and osteoblasts [2]. Support for this hypothesis comes from studies of *peroxisome proliferators-activated receptor-γ* (*Pparγ*), a master regulator of adipocyte differentiation, deficient embryonic stem cells that showed an increase in osteoblast differentiation [3]. In contrast, calvarial adipocyte differentiation is augmented when *runt-related transcription factor 2 (Runx2)*, a master regulator of osteoblast differentiation has been knocked out [4]. Transcription factors Runx2 and *Sp7 transcription factor 7* (*Sp7*) regulate MSC commitment to osteoblast differentiation along with bone morphogenetic protein (BMP) signaling pathway [5]. Conversely, Pparγ and CCAAT/enhancer binding protein (C/EBP) transcription factor family members drive MSCs differentiation toward adipocytes [6]. Other proteins that regulate the balance between adipocyte and osteoblast differentiation are tafazzin, Wnt5a, Wnt10b, Msx2, C/EBPβ and basic helix-loop-helix (bHLH) family member e40 (Bhlhe40) [6]–[8]. Aforementioned transcription factors suppress adipocyte differentiation and promote osteoblast differentiation. Regardless of these studies, the precise molecular mechanisms that regulate the balance between osteoblast and adipocyte differentiation in the bone marrow has yet to be elucidated. Hence, we aimed to identify transcription factors that regulate the direction of differentiation toward osteoblast or adipocyte by analyzing their genome-wide expression profiles in differentiation time series experiments.

We noticed in early phases a subgroup of transcription factors that appeared to function in both osteoblasts and adipocytes differentiation. Particularly, bHLH superfamily transcription factors were significantly enriched and up-regulated in the early phase of osteoblast differentiation.

The bHLH superfamily comprises transcription factors that form homo- or heterodimers and typically bind to a consensus sequence (CANNTG) called an E-box [9]. It is well known that bHLH transcription factors play important roles in development and cell differentiation. For example, Myod1 is a key differentiation factor of myoblasts and Srebf1 is involved in adipocyte differentiation [10], [11]. Hairy and enhancer of split (Hes) family members of bHLH superfamily are crucial regulators of cortical development [12].

Here, we have identified Inhibitor of DNA binding 4 (Id4), which also belongs to the bHLH superfamily as a key molecule that regulates the direction of differentiation toward osteoblast or adipocyte *in vitro* and *in vivo* using genome wide expression study. Furthermore, we established that Id4 promotes osteoblast differentiation by enhancing Runx2 transcriptional activity through stabilization of Runx2 protein. The new role of Id4 in directing osteogenic and adipogenic cell fate makes it a likely target for preventing the onset of senile osteoporosis.

## Results

### Genome-wide expression profile predicts *Id4* as a candidate molecular switch in osteoblast and adipocyte differentiation

**Figure 1:**
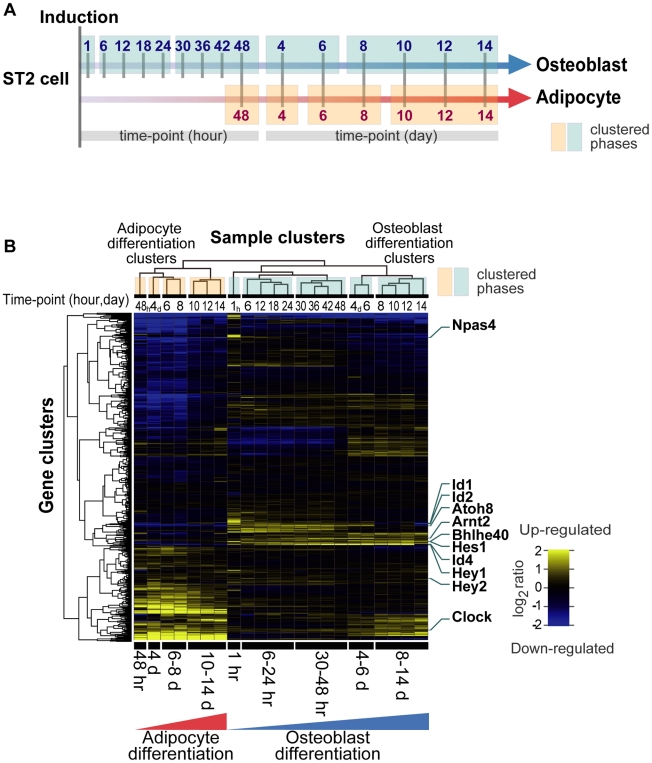
Transcription factor gene expression framework of differentiating ST2 cells. (A) Time-course sampling for gene expression analyses in ST2 cells. Vertical bars crossing the gradated blue and orange arrows indicate the sampling times during osteoblast and adipocyte differentiation, respectively. Five blue (osteoblasts) and four orange (adipocytes) boxes indicate the hierarchical clustering-derived phases of differentiation. (B) Gene expression heat map and clustering results of 1,270 transcription factors in 22 time-course samples.

**Figure 2:**
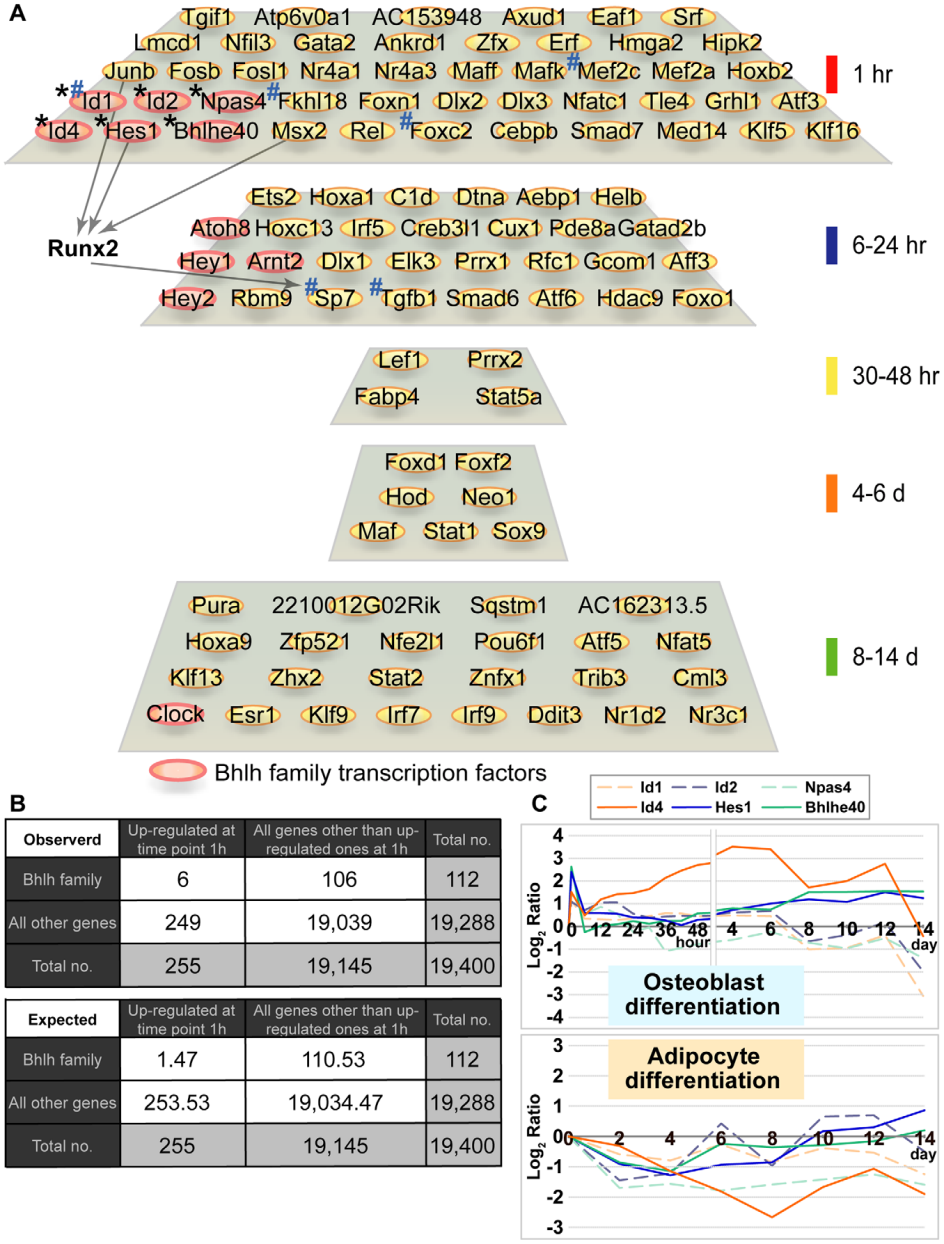
Enrichment of bHLH family in early phase of osteoblast differentiation and their expression pattern. (A) Summary of up-regulated transcription factors (Figure S1) associated with five phases of osteoblast differentiation. Asterisk (*): bHLH transcription factors that are overrepresented only during the early phase of osteoblast differentiation (*p*<0.01, Figure 2B). Sharp (#): transcription factors known to be associated with osteoblast differentiation and/or bone development. (B) Chi-square (χ^2^) test for independence of bHLH superfamily genes in the immediate early phase (1 hr) of osteoblast differentiation. Null hypothesis: the occurrence of up-regulated bHLH transcription factors observed during the immediate early phase of osteoblast differentiation among all genes associated with probe set of Affymetrix GeneChip Mouse Genome 430 2.0 array is independent. Results of the contingency table (2×2 cells, degree of freedom = 1) allowed to reject the Null hypothesis. The expression of bHLH transcription factors was significantly up-regulated during the immediate early phase of osteoblast differentiation (*p*<0.01). (C) Expression pattern of six bHLH family genes in osteoblast and adipocyte differentiation. The chart represents expression changes of six bHLH genes during osteoblast and adipocyte differentiation. The horizontal axis indicates each time point after induction. The vertical axis indicates the logarithmic expression ratio (base 2) of each time point in comparison to the control (0 day).

To delineate the sequential changes of transcription factors activating and repressing downstream osteogenic and/or adipogenic target genes, we evaluated the differentiation capability toward both osteoblasts and adipocytes using six cell lines (ST2, C2C12, DFAT-D1, PA6, 10T1/2, NRG). Of these, bone marrow-derived stromal cell line ST2 differentiated most efficiently into both osteoblasts and adipocytes (data not shown). Using Affymetrix mouse GeneChip, we aimed to identify clusters of transcription factors that are temporally co-regulated in one but not in another cluster (CIBEX Accession number: CBX90). Of 1,270 transcription factors, 407 genes were significantly up- or down- regulated in either osteoblast or adipocyte differentiation compared to the non-induced control (Table 1 and Table S1). Hierarchical clustering analysis of transcription factor gene expression data at 15 osteoblast and seven adipocyte differentiation time points (Figure 1A) revealed distinct clusters that represent phases of sequentially expressed transcription factors (Figure 1B). Differentiation into osteoblasts is characterized by five phases (Figure S1) whereas adipocyte differentiation resulted in four phases (Table S1). The early phases of osteoblast (1 hr) and adipocyte (48 hr) differentiation showed the greatest variability in transcription factor expression levels (Figure 2A and Table S1).

**Table 1:** Expression behavior of 1,270 transcription factors selected from all mouse genes (Ensembl release 52) based on GO IDs (Table 3) during osteoblast and adipocyte differentiation.

Transcription Factors		Osteoblast Differentiation				
		↑	↓	↑↓	≈	Total
Adipocyte Differentiation	↑	22	6	4	70	102
	↓	25	40	21	132	218
	↑↓	3	5	2	2	12
	≈	42	26	7	863	938
	Total	92	77	34	1,067	1,270

Arrows indicate up- (↑), down-regulated (↓), or up- and down-regulated (↑↓) transcription factors. No change in expression is symbolized by the almost equal sign (≈).

Chi-square testing for over-representation of transcription factors in each differentiation phase supported only six up-regulated bHLH superfamily members (*Id1*, *Id2*, *Npas4*, *Id4*, *Hes1* and *Bhlhe40*) of the immediate early phase osteoblast differentiation (1 hr) as significantly (*p*<0.01) enriched (Figure 2B). Since *Id4*, *Hes1* and *Bhlhe40* expression increased (decreased) twofold or greater during osteoblast (adipocyte) differentiation compared to the control (Figure 2C and Table 2), these transcription factors are likely to play a pivotal role in the regulation of osteoblast and adipocyte differentiation.

**Table 2:** Expression of eleven bHLH transcription factors shown in Figure 2A.

	Differentiation		Differentiation Clusters	
Gene Symbol	Osteoblast	Adipocyte	Osteoblast	Adipocyte
***Bhlhe40***	**↑**	**↓**	1h ↑	4d ↓
***Hes1***	**↑**	**↓**	1h ↑	4d ↓
*Id1*	↑↓	↓	1h ↑	10d–14d ↓
*Id2*	↑↓	↓	1h ↑	2d ↓
***Id4***	**↑**	**↓**	1h ↑	4d ↓
*Npas4*	↑↓	↓	1h ↑	2d ↓
*Arnt2*	↑	≈	6h–24h ↑	≈
*Atoh8*	↑↓	↓	6h–24h ↑	2d ↓
*Hey1*	↑	≈	6h–24h ↑	≈
*Hey2*	↑	≈	6h–24h ↑	≈
*Clock*	↑	↑	8d–14d ↑	10d–14d ↑

Of eleven transcription factors, *Bhlhe40*, *Hes1* and *Id4* were two-fold or greater up-regulated during osteoblast differentiation. On the other hand, these genes were one-half fold or greater down-regulated during adipocyte differentiation. Arrows indicate up- (↑), down-regulated (↓), or up- and downregulated (↑↓) bHLH genes. No change in expression is symbolized by the almost equal sign (≈). Crosstalk functions between osteoblast and adipocyte differentiation have been reported for Bhlhe40 [8], Hes1 [13], [14] and Id4 (this study), only.

Indeed, Hes1 and Bhlhe40 are known to be involved in both differentiation pathways [8], [13], [14], whereas Id4 has not yet been implicated in either differentiation pathways. Additionally, *Id4* expression patterns in osteoblast and adipocyte differentiation were also compared by quantitative real-time PCR (qRT-PCR). Expression of *Id4* significantly increased during osteoblast differentiation, attained a peak on day 4 and decreased thereafter (Figure S2A). In contrast, *Id4* expression decreased during adipocyte differentiation (Figure S2B). Expression levels of *Id1* and *Id2* were also up-regulated in the early stage (1 hr) of osteoblast differentiation, but thereafter their expression dropped to base levels (Figure 2C, upper panel). Therefore, we hypothesized that Id4 may act as a novel molecular switch in osteoblast and adipocyte differentiation.

Aside from *Id4*, *Hes1* and *Bhlhe40*, we identified additional bHLH members and various hypothetical and non-bHLH transcription factors as phase-specific candidate regulators of osteo- and adipogenesis (Figure 2A and Table S1). The genes listed in Table S1 await further functional characterization regarding their involvement in osteoblast and/or adipocyte differentiation.

### *Id4* knockdown suppresses osteoblast differentiation of MSCs, and overexpression promotes osteoblast differentiation

**Figure 3:**
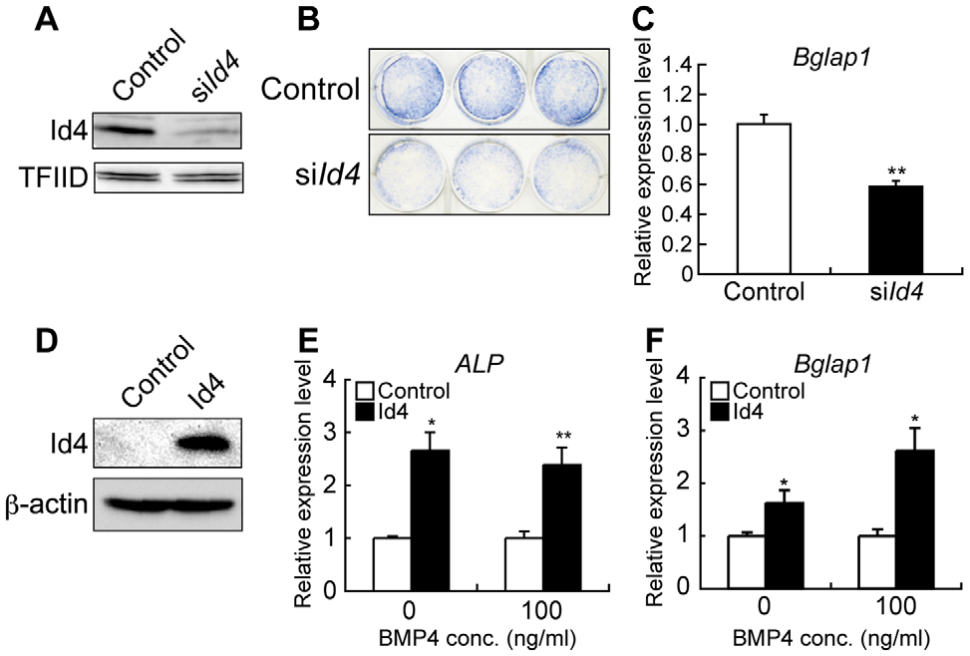
*Id4* promotes osteoblast differentiation in ST2 cells. (A) Western blotting of anti-Id4 antibody using ST2 nuclear extract. TFIID was detected as a loading control. Specific reduction of Id4 expression at day 2 after induction of ST2 cells treated with *Id4* siRNA (si*Id4*) for osteoblast differentiation. (B,C) Decrease in ALP activity (B) and *Bglap1* expression (C) at day 6 after induction of osteoblast differentiation in ST2 cells treated with si*Id4* as compared to the negative control. (D) Western blotting of anti-Id4 antibody using ST2 cell lysate, infected with Id4 retrovirus or control retrovirus (control). β-actin was detected as a loading control. (E,F) Increase in *ALP* (E) and *Bglap1* (F) expression at day 4 after infection with *Id4* virus as compared to the control virus in non-induced ST2 and ST2 osteoblast. Relative expression levels of *ALP* and *Bglap1* mRNA were measured by qRT-PCR. qRT-PCR data were subjected to Student's t-tests. Each error bar represents the mean ± SE of triplicates. *p<0.05 versus control, **p<0.01 versus control.

To evaluate the potential role of Id4 in ST2 osteoblast differentiation, Id4 was suppressed by siRNA knockdown. As shown in Figure 3A, *Id4* siRNA (si*Id4*)-treated ST2 cells differentiating into osteoblasts showed a significant decrease in Id4 expression. The decline of *Id4* expression was accompanied by weak alkaline phosphatase (ALP) activity and reduced *bone γ carboxyglutamate protein 1* (*Bglap1* also called *osteocalcin*) expression (Figure 3B and 3C). Since both are markers of osteoblast differentiation, it appeared that Id4 is important in osteoblast differentiation of MSCs. We next evaluated whether forced expression of Id4 can promote osteoblast differentiation in MSCs by retroviral systems (Figure 3D). ST2 cells infected with Id4 recombinant retrovirus showed increased expression levels of *ALP* and *Bglap1* compared to cells infected with control virus independent of the presence or absence of BMP4 (Figure 3E and 3F). Taken together, Id4 promotes osteoblast differentiation of MSCs.

### *Id4* knockdown stimulates adipocyte differentiation, and overexpression attenuates lipid accumulation

**Figure 4:**
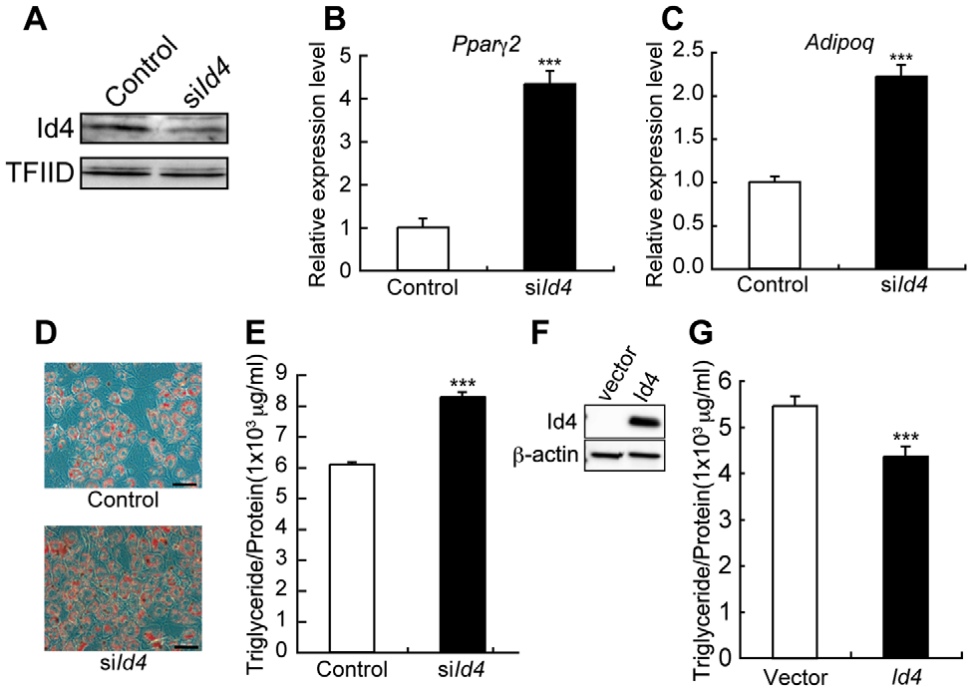
*Id4* weakly suppresses adipocyte differentiation in ST2 cells. (A) Western blotting of anti-Id4 antibody using ST2 nuclear extract. TFIID was detected as a loading control. Specific reduction of Id4 expression at day 2 after induction of ST2 cells treated with si*Id4* for adipocyte differentiation. (B,C) Increase in *Pparγ2* (B) and *Adipoq* (C) expression at day 1 after induction of adipocyte differentiation and treatment with si*Id4*. Relative expression levels of *Pparγ2* and *Adipoq* mRNA were measured by qRT-PCR. (D) Oil Red O staining of ST2 cells at day 4 after induction of adipocyte differentiation in presence of control or si*Id4*. Original magnification: ×200; bar = 100 µm. (E) Lipid content of ST2 cells measured as triglyceride level at day 5 after adipogenic induction in presence of control or si*Id4*. (F) Western blotting of anti-Id4 antibody using Cos7 cell lysate, transfected with Id4 expression vector or empty vector (Vector) as a control. β-actin was detected as a loading control. (G) Lipid content of ST2 cells transfected with Id4 expression vector or empty vector measured as triglyceride level at day 5 after adipogenic induction. qRT-PCR and lipid content data were subjected to Student's t-tests. Each error bar represents the mean ± SE of triplicates. *p<0.05 versus control and ***p<0.005 versus control.

*Id4* knockdown in adipogenesis-induced ST2 cells (Figure 4A) significantly increased expression levels of other adipogenic marker genes such as *Pparγ2* [15] and *Adipoq* [16] (Figure 4B and 4C). Concomitantly, the number of Oil Red O stained lipid droplets and triglyceride levels also increased (Figure 4D and 4E). Forced expression of Id4 was confirmed by transfection into Cos7 cells with Id4 expression vector (Figure 4F). Adipocytes differentiated from ST2 cells transfected with Id4 expression vector showed slightly but significantly decreased lipid accumulation compared to empty vector transfectants (Figure 4G). The combined results of *Id4* siRNA knockdown and overexpression in ST2 suggest that Id4 attenuates differentiation of MSCs into adipocytes.

### *Id4*-knockout mice (*Id4^−/−^*) showed impaired osteoblast differentiation

**Figure 5:**
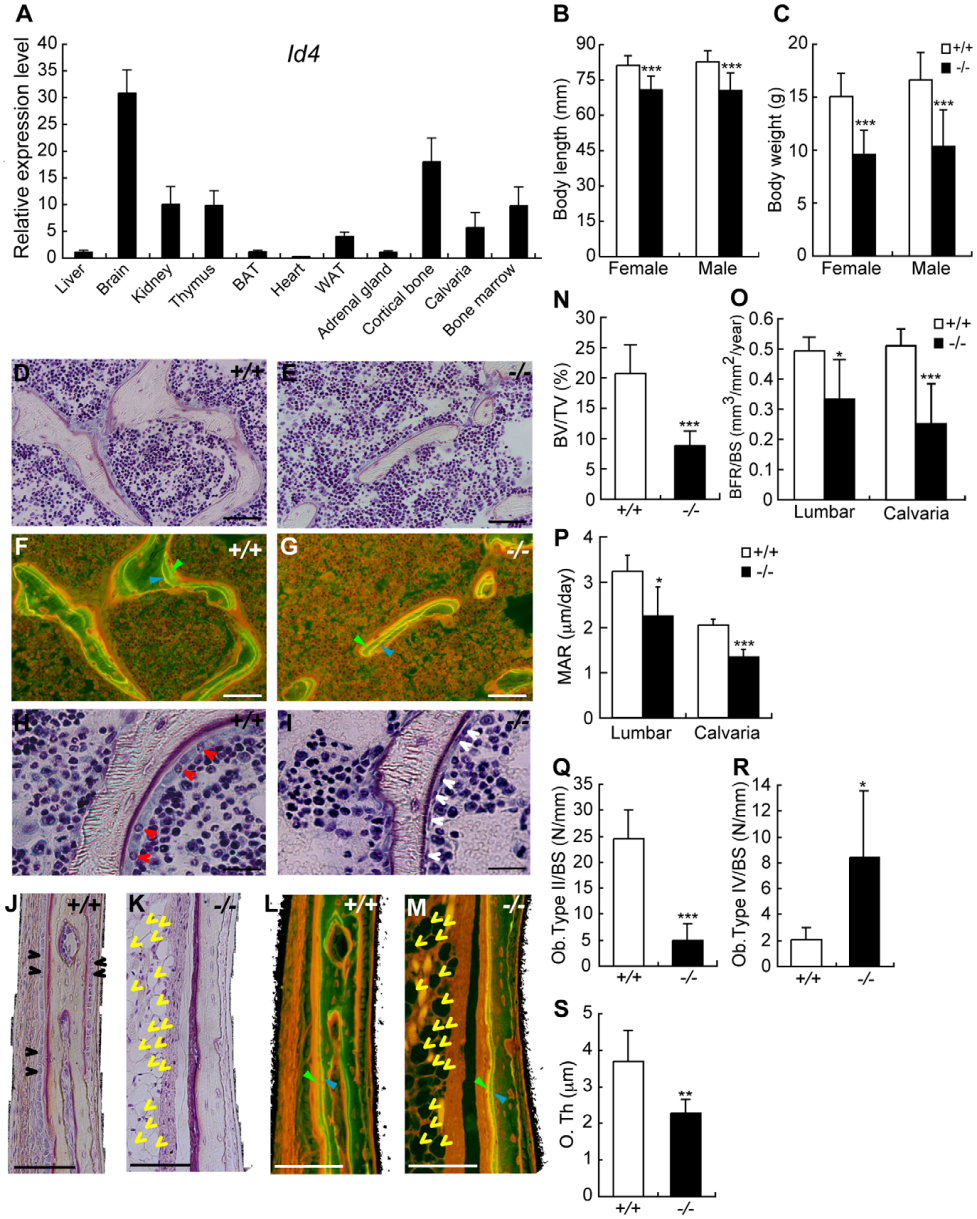
4-week-old *Id4* knockout (*Id4* ^−/−^) mice show disturbance in growth and impaired osteoblast differentiation. (A) Tissue distribution of *Id4* mRNA in C57BL/6J male mice was measured by qRT-PCR. Brown adipose tissues; BAT, White adipose tissues; WAT. (B,C) Body length (B) and body weight (C) of wild-type (*Id4* ^+/+^) and *Id4* ^−/−^ mice. (D,E,H,I) Villanueva staining of the 6^th^ lumbar bone of *Id4* ^+/+^ mice (D,H) and *Id4* ^−/−^ mice (E,I). Red and white arrowheads indicate active cuboidal-shaped osteoblasts (type II osteoblasts) and flattened resting osteoblasts (type IV osteoblasts), respectively. (F,G,L,M) Bone formation rate (BFR) in the lumbar regions (F,G) and the lateral calvarial bone (L,M) of *Id4^+/+^* (F,L) and *Id4^−/−^* (G,M) mice. The BFR was measured using fluorescence microscopy following double-staining with tetracycline hydrochloride (blue arrowhead) and calcein (green arrowhead). Yellow arrowheads indicate adipocytes. (J,K) Villanueva staining of the lateral calvarial bone of *Id4* ^+/+^ (J) and *Id4* ^−/−^ mice (K). Black arrowheads indicate osteoblasts. The original magnifications and scale bars of the images are ×400 and 50 µm (D–G), ×400 and 30 µm (H,I) and ×200 and 50 µm (J–M). (N) Bone volume (BV) to total volume (TV) ratio in the 6^th^ lumbar bone. (O,P) BFR to bone surface (BS) ratio (O) and mineral apposition rate (MAR) (P) in the 6^th^ lumbar bone and lateral calvarial bone. (Q,R) Number of type II osteoblasts (Q) and type IV osteoblasts (R) on the corresponding area of the lumbar BS. (S) Osteoid thickness (O. Th) in the lateral calvarial bone. N-S: *Id4* ^+/+^ (n = 6), *Id4* ^−/−^ (n = 6). All data were subjected to Student's t-tests. Each error bar represents the mean ± SE. *p<0.05 versus control, **p<0.01 versus control and ***p<0.005 versus control.

Previously *Id4* ^−/−^ mice were studied only in context of neural development [17]. We confirmed that *Id4* expression was highest in the brain followed by cortical bone, kidney, thymus and bone marrow of C57BL/6J mice (Figure 5A). The body length and weight of 4 weeks old *Id4* ^−/−^ mice was 13–15% shorter and 35–40% lower compared to wild-type (*Id4^+/+^*) littermates (Figure 5B and 5C). *Id4* ^−/−^ mice showed severe growth retardation and died by 5 weeks. In addition, we observed visible skeletal phenotypes of *Id4^−/−^* mice, but no skeletal deformities (data not shown). Altogether, our data hint at an important role of Id4 in bone formation.

Bone histological analysis of *Id4^−/−^* mice revealed significantly decreased bone volume (BV) in the 6^th^ lumbar (Figure 5D and 5E). The bone formation rate (BFR) was decreased in *Id4* ^−/−^ mice compared to *Id4^+/+^* mice (Figure 5F and 5G). The BV to total volume ratio, BFR to bone surface (BS) ratio and mineral apposition rate (MAR) of *Id4* ^−/−^ mice were 57.3%, 28.1% and 30.7% lower compared to *Id4* ^+/+^ mice in the 6^th^ lumbar, respectively (Figure 5N–5P).

In *Id4* ^+/+^ mice, active cuboidal-shaped osteoblasts (type II osteoblasts) were distributed in a row along the lumbar BS (Figure 5H), whereas in the corresponding region of *Id4* ^−/−^ mice osteoblasts were predominantly flat and resting (type IV osteoblasts; lining cells) (Figure 5I). The number of osteoblasts as a whole did not change significantly between *Id4^+/+^* and *Id4^−/−^* mice (data not shown). However, in *Id4^−/−^* mice the population of active osteoblasts was reduced (type II, Figure 5Q), whereas inactive osteoblasts accumulated (type IV, Figure 5R). These findings imply that Id4 modulates both differentiation of osteoblasts from pre-osteoblasts and regulation of osteoblast maturation.

Impaired bone formation was also observed in the lateral calvaria of *Id4^−/−^* mice (Figure 5K and 5M). In wild type mice, osteoblasts were closely lined up along the calvarial BS (Figure 5J). In contrast, no osteoblasts were observed along the calvarial bone of *Id4^−/−^* mice (Figure 5K). The osteoid thickness, BFR to BS ratios and MAR of *Id4* ^−/−^ mice calvarial bones were 61.5%, 49.1% and 65.2% of *Id4* ^+/+^ mice, respectively (Figure 5S, 5O, and 5P). These results suggest that Id4 is important for both endochondorial and membranous ossification. Growth Plate Width and Longitudinal Growth Rate (Lo. G. R) of *Id4* ^−/−^ mice tibia were 68.4% and 57.1% of *Id4* ^+/+^ mice, respectively (Figure S3A, S3B, S3C, S3D), which may have caused the growth retardation of *Id4* ^−/−^ mice.

### Id4 interacts with Hey2 and inhibits the transcriptional repression of Hey2

**Figure 6:**
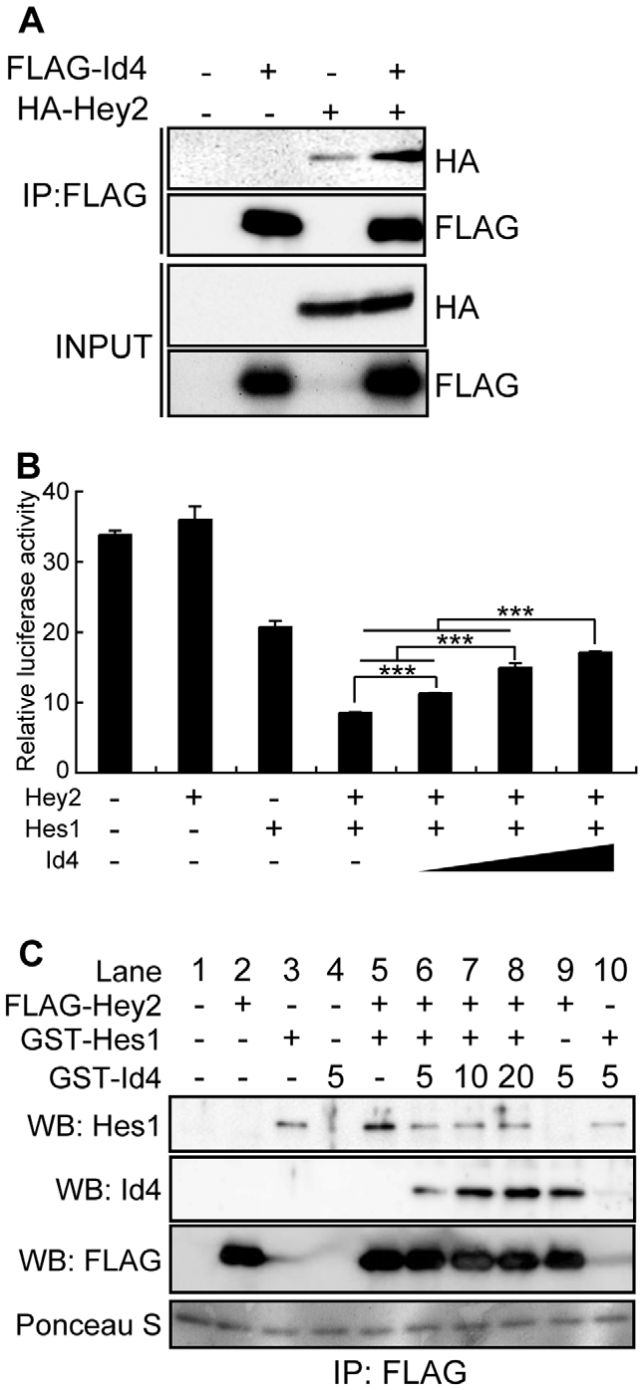
Id4 interacts with Hey2 and inhibits the suppression of reporter activity by Hey2/Hes1 complex. (A) Interaction of FLAG-tagged Id4 (FLAG-Id4) and human influenza hemagglutinin-tagged Hey2 (HA-Hey2) was confirmed in 293FT cells by immunoprecipitation (IP). (B) CV1 cells transfected with 6×E-box luciferase reporter, Hey2 and Hes1 expression vectors and with increasing doses of Id4 expression vector. Luciferase assay data were subjected to Student's t-tests. Each error bar represents the mean ± SE of triplicates. ***p<0.005 versus control. (C) *In vitro* assay of Id4 dose-dependent attenuation of Hey2-Hes1 complex interaction. GST-Id4 volume added was 5, 10 and 20 µl, respectively. WB: Western blotting.

Id family members are known to heterodimerize with other bHLH transcriptional factors, thus inhibiting the binding to the E-box motif [18]. To explore whether heterodimerized Id4 switches the direction of osteoblast and adipocyte differentiation, we assayed Id4 protein-protein interactions and analyzed their effects. Using immunoprecipitation we attempted to capture for candidate bHLH transcription factors that bind to Id4. Out of four tested bHLH transcription factors (Hes1, hairy/enhancer-of-split related with YRPW motif 1; Hey1, hairy/enhancer-of-split related with YRPW motif 2; Hey2 and Bhlhe40) (Figure S4A), only Hey2 bound to Id4 (Figure 6A, Figure S4A and S4B). An earlier study demonstrated that Hey2 is forming heterodimers with Hes1, which then bind to the E-box motif and repress transcription [19]. Therefore, we tested the effect of Id4 on transcriptional repression of Hey2/Hes1 heterodimers against the E-box element. Transcriptional repression onto E-box element in the presence of either Hey2 or Hes1 showed no effect or 39% inhibition of E-box transcriptional activity relative to control (empty expression vector), respectively (Figure 6B). Although luciferase activity was lowest in the presence of both Hey2 and Hes1, inhibition of transcriptional repression by Id4 increased in dose-dependent manner (Figure 6B). We also demonstrated that Hey2-Hes1 binding was abrogated with the dose-dependent increase of Id4 (Figure 6C, lane 5 and lane 6). Taken together, we confirmed that Id4 reverses the transcriptional repression by Hey2-Hes1 heterodimer in a dose-dependent manner.

### Id4/Hey2 complex indirectly enhances Runx2 transcriptional activity

**Figure 7:**
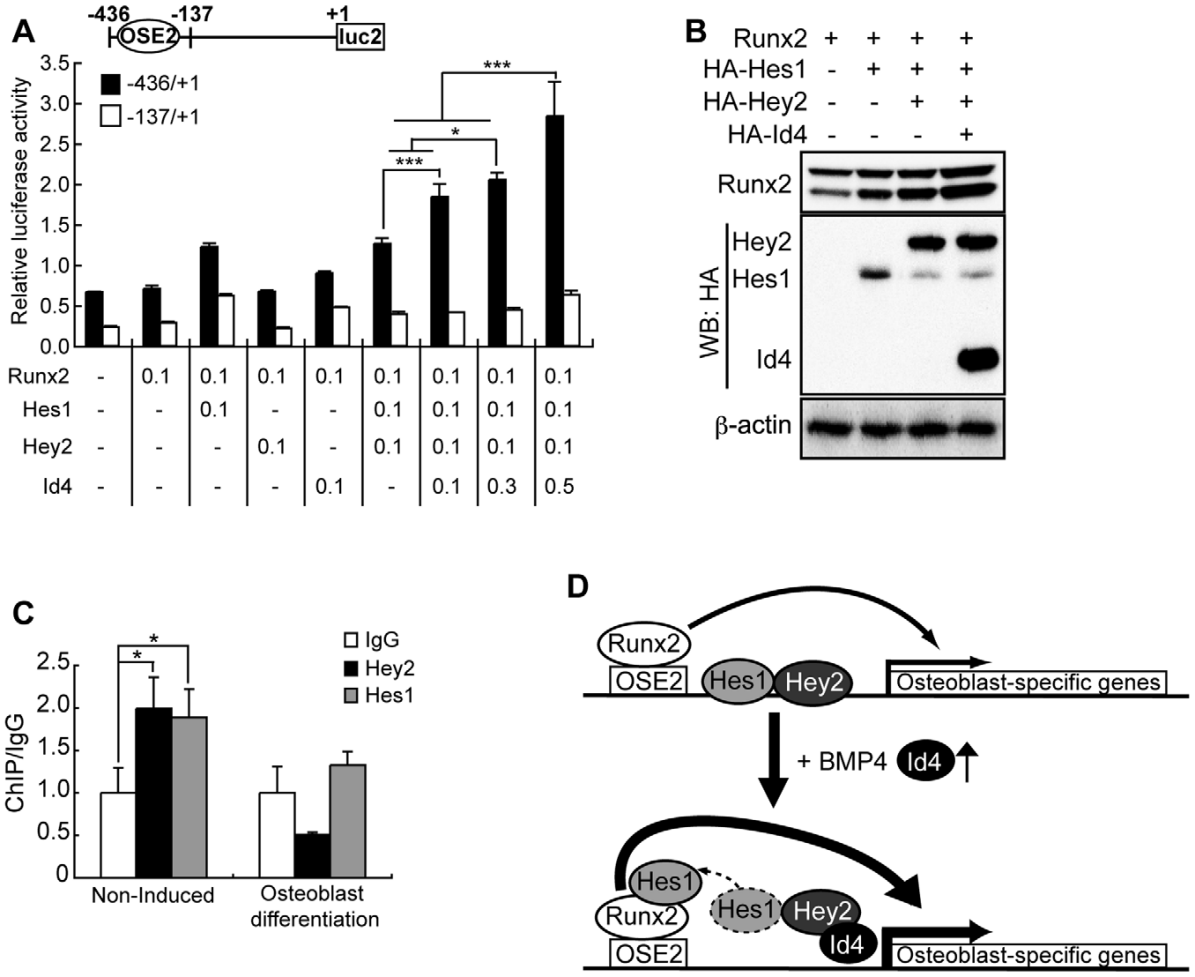
Id4 enhanced Runx2 transcriptional activity through stabilization of Runx2 protein. (A) Relative luciferase activity of *Bglap1* promoter in CV1 cells transfected with Runx2, Hes1, Hey2 and Id4 expression vectors. *Bglap1* promoter luciferase reporters were used as Runx2-dependent (−436bp/+1bp) or Runx2-independent (−137bp/+1bp). Luciferase assay data were subjected to Student's t-tests. Each error bar represents the mean ± SE of triplicates. *p<0.05 versus control and ***p<0.005 versus control. +1; transcription start site, bp; base pair. (B) Western blot analysis of Cos7 whole cell lysate using anti-Runx2, anti-HA and anti-β-actin (loading control) antibodies. The stability of Runx2-Hes1 complex increased in the presence of Id4. (C) ChIP-qPCR analyses of Hey2 and Hes1 binding onto *Bglap1* promoter in ST2 cells at day 4 after induction of osteoblast differentiation or non-induced ST2 cells. (D) A model of Id4 promoting Runx2-induced osteoblast differentiation.

The presence of inactive osteoblasts (Figure 5I and 5R) in the bone tissues of the *Id4^−/−^* mice let us assume that Id4 may affect the actions of Runx2 and Sp7, a key osteogenic differentiation molecule [20]–[22]. Osteoblast marker gene *Bglap1* expression level decreased not only in primary osteoblasts but also in embryonic day18.5 (E18.5) limb of *Id4^−/−^* mice (Figure S5A and S5B). *Bglap1*, a target gene of Runx2 has an E-box element other than osteoblast-specific element 2 (OSE2), which binds Runx2 to the promoter [23]. Therefore, we measured the promoter activity to examine the influence of Id4 on *Bglap1* E-box promoter-dependent transcriptional activity of Runx2. Although the suppression of *Bglap1* promoter activity by addition of Hes1 and Hey2 was not detected, a dose-dependent increase of transcriptional activity by Id4 was observed when testing the OSE2 element-containing promoter. When using the promoter without OSE2 the increase in transcriptional activity was not seen (Figure 7A). Since direct interaction between Id4 and Runx2 was ruled out experimentally (data not shown), Id4 may indirectly influence Runx2 transcriptional activity through Hes1. Hes1 is known to stimulate the transcriptional activity of Runx2 protein by increasing its stability during osteoblast differentiation [24]. We also confirmed that the addition of Hes1 stabilizes Runx2 protein. Interestingly, the addition of Id4 further increased the stabilization and accumulation of Runx2 (Figure 7B). Taken together, it appears that Id4 enhances Runx2 transcriptional activity through stabilization of Runx2 protein.

Taking into account the timing of the elevated *Id4* expression (Figure 2C and Figure S2A), our results strongly suggest that Id4 is indirectly driving the Hes1-mediated Runx2 stabilization during osteoblast differentiation. The facts that both Hes1 and Hey2 bind to the OSE2 element-containing *Bglap1* promoter region assessed by ChIP-qPCR, and that the amount of bound Hes1 and Hey2 decreased in ST2 osteoblasts (Figure 7C) further support this idea. However, the exact binding site of Hes1-Hey2 heterodimer remains to be identified. The proposed mechanism of Id4 action during osteoblast differentiation is illustrated in Figure 7D.

### *Id4^−/−^* mice showed increased adipocytes

**Figure 8:**
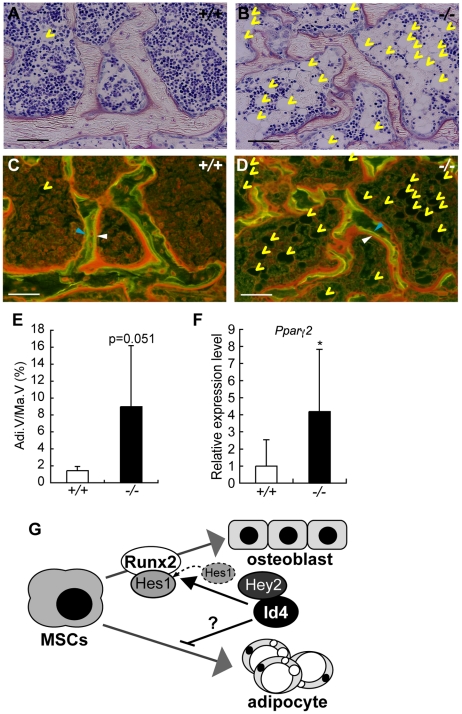
4-week-old *Id4* ^−/−^ mice show increased adipocytes in bone marrow. (A,B) Villanueva staining of epiphyseal tibia of *Id4* ^+/+^ (A) and *Id4* ^−/−^ mice (B). (C,D) The BFR was measured in the tibial epiphysial regions in *Id4* ^+/+^ (C) and *Id4* ^−/−^ mice (D) using a fluorescence microscopy following double-staining with tetracycline hydrochloride (blue arrowheads) and calcein (white arrowheads). Yellow arrowheads indicate adipocytes. The original magnifications and scale bars of the images are ×400 and 100 µm. (E) The total area occupied by adipocytes (Adi.V) in the epiphyseal tibia bone marrow. *Id4* ^+/+^ (n = 6), *Id4* ^−/−^ (n = 6). (F) *Pparγ2* mRNA expression is significantly elevated in bone marrow cells of *Id4* ^−/−^ compared to *Id4* ^+/+^ mice. Relative expression levels of *Pparγ2* mRNA were measured by qRT-PCR. *Id4* ^+/+^ (n = 9), *Id4* ^−/−^ (n = 10). All data were subjected to Student's t-tests. Each error bar represents the mean ± SE. *p<0.05 versus control. (G) The role of Id4 in bone formation and bone marrow environment.

*Id4* knockdown promoted adipocyte differentiation in ST2 cells (Figure 4B–4E). Histological analysis of *Id4^−/−^* tibia bones revealed elevated numbers of adipocytes in epiphyseal bone marrow of tibia compared to *Id4^+/+^* mice (Figure 8A–8D). The entire analyzed area of epiphyseal tibia bone marrow was occupied by adipocytes in *Id4* ^−/−^ mice (Figure 8E). Moreover, *Pparγ2* expression levels were also increased in bone marrow cells of femur and tibia of *Id4* ^−/−^ mice (Figure 8F). In comparison to *Id4^+/+^* mice, the number of adipocytes in the lateral calvaria was markedly increased in *Id4* ^−/−^ mice (Figure 5K). These aberrant traits observed in *Id4^−/−^* mice implicate Id4 as a crucial molecule in the lineage choice of MSCs differentiating into either osteoblasts or adipocytes (Figure 8G).

## Discussion

In this study, we have delineated clusters of transcription factors that act as key regulators in the osteoblast and adipocyte differentiation network. The observation of sometimes disparately regulated transcription factors, led us to hypothesize a molecular switch function that promotes lineage-specific differentiation (e.g. osteoblast differentiation) and inhibits alternative differentiation pathways (e.g. adipocyte differentiation). To substantiate the hypothesis, we clustered gene expression profiles during osteoblast/adipocyte differentiation and identified Id4 as a candidate regulator of cell lineage choice. Id family members Id1–4 also belong to the bHLH superfamily, but lack the DNA binding domain. Heterodimerization of Id proteins with other bHLH proteins facilitates dominant negative regulation. A study by Bedford *et al*. using *Id4* ^−/−^ mice established Id4 as a regulator of proliferation and differentiation of neural precursor cells [17]. Until now, however, bone and skeletal abnormalities of this mouse model have not been reported. Besides our report, expression profile studies of BMP-independent osteoblast differentiation of human MSCs and BMP2-induced osteoblast differentiation using calvarial cells derived from Runx2-deficient mice [25], [26] demonstrated that the expression level of *Id4* gene increases during osteoblast differentiation. These results suggest that Id4 plays an important role during osteoblast differentiation. Yet, the precise mechanism of Id4 action and regulation remained enigmatic. We have clearly demonstrated by *in vitro* and *in vivo* loss-of-function analysis that Id4 enhances osteogenic differentiation. Furthermore, we established a model of Id4 playing the role of molecular switch in osteoblast differentiation of MSCs (Figure 7D). Id4 expression increases upon BMP-induced osteoblast differentiation. Accumulating Id4 proteins transiently interact with Hes1-bound Hey2, thus triggering the release of Hes1 and the formation of Id4-Hey2 heterodimers and Hes1-Runx2 complexes. The binding of Hes1 to Runx2 potentiates the transcriptional activity of Runx2 and therefore osteoblast differentiation.

Hes1 and Hey2 are the target molecules of Notch signaling [19]. Until now, the relationship between Notch and BMP signaling pathways has been characterized by conflicting reports. On one hand experiments using ST2 cells showed that Notch1 suppresses the BMP-induced differentiation into osteoblasts [27]. On the other hand, Nobta et al. [28] reported that Notch signaling enhances BMP-induced osteoblast differentiation of C2C12 or MC3T3-E1 cells. At this point, there are insufficient data to resolve the controversy about the role of Notch signaling in BMP-induced osteoblast differentiation. However, our model (Figure 7D) may help to clarify the function of Notch signaling. In the absence of Id4, Hes1-Hey2 heterodimer just occupies the promoter region of the Runx2 target gene, whereas in the presence of Id4, Id4-Hey2 and Hes1-Runx2 complexes increase simultaneously. The concomitant increase of both complexes enhances the transcriptional activity of Runx2. Thus, we propose that availability and concentration of Id4 might account for the disparate roles of Notch signaling.

In *Id4^−/−^* mice, the drop of calvarial BFR is consistent with the phenotype of *Id1*/*Id3* heterozygous knockout mice. After BMP stimulation, *Id1/Id3* heterozygous knockout mice-derived calvarial cells showed reduced proliferation activity compared to calvarial cells derived from wild-type mice [29]. Thus, the decreased rate of calvarial bone formation in *Id4^−/−^* mice might be the consequence of reduced osteoblast proliferation. The expression levels of *Id1*and *Id2* were also up-regulated in the early stage of BMP4-induced osteoblast differentiation (Figure 2A). Indeed, it has been reported that *Id1* is an important early gene in osteoblasts after BMP stimulation [30], [31]. Although the biological significance of Id1 in the regulation of MSCs has to be elucidated in future studies, in ST2 cells, expression levels of *Id1* and *Id2* immediately returned to base levels (Figure 2C). In contrast, *Id4* expression levels in ST2 and 3T3-E1 cells continued to rise until 4 days (Figure 2C and Figure S2A) and 7days, respectively [32].

Systemic hormones and local cytokines are known to be central regulators of bone formation. Serum levels of growth hormone (IGF1) and thyroid hormones (T3 and T4) did not change significantly between *Id4* ^+/+^ and *Id4* ^−/−^ mice (data not shown). Hence, impaired bone formation is most likely independent of hormonal factors and caused by repression of osteoblast differentiation.

Since the expression level of *Id4* decreases during adipocyte differentiation (Figure 2C and Figure S2B), Id4 was believed to inhibit MSCs differentiation into adipocytes. We demonstrated that Id4 suppression promoted adipocyte differentiation (Figure 4B–4E and Figure 8A–8E), but Id4 overexpression slightly decreased lipid accumulation level in ST2 adipocytes (Figure 4G). In an effort to shed light on the molecular mechanism, we assayed the expression level of *Pparγ2*, a master regulator of adipocyte differentiation. *Pparγ2* expression increased in adipogenic-induced ST2 cells when *Id4* was knocked down (Figure 4B) and in bone marrow cells of femur and tibia of *Id4^−/−^* mice (Figure 8F). However, the results of luciferase reporter assays ruled out effects of Id4 on the promoter activity of *Pparγ2* (data not shown). *Pparγ2* down-regulation by Id4 might involve a yet unknown, indirect regulatory mechanism. We noticed that the number of osteoclasts increased in the tibial epiphyseal regions and the 6^th^ lumbar vertebra (data not shown). In view of an earlier report on Pparγ promoting osteoclast differentiation by activating *c-fos* [33], we interpret the increased number of adipocytes and osteoclasts in 6^th^ lumbar and tibial bone of *Id4* ^−/−^ mice as a result of indirect activation of *Pparγ1* and/or *Pparγ2* transcriptional activity by lack of Id4. To corroborate these assumptions, additional analyses of the relationships and interactions of Id4, Pparγ and c-fos in context of adipocyte and osteoclast differentiation are necessary.

In summary, delineating clusters of transcription factors is a powerful strategy to identify cell fate-determining members of regulatory networks. Concerted application of our genome-wide expression profiling analyses and validation of transcription factor candidate regulators synthesize knowledge of specific molecular mechanism underlying osteoporosis and/or metabolic disease. In case of Id4, our findings reflect the potential pen-ultimate position of Id4 in the Runx2 activation/repression, which permits the differential integration of various upstream signals. Since BMP and Notch signaling affect osteoblast differentiation at different phases of differentiation, modulation of Id4 expression may create new venues for treating the onset of osteoporosis.

## Materials and Methods

### Cell culture

ST2 cells were obtained from RIKEN BioResource Center (BRC, Tsukuba, Japan) and cultured as described [34]. Primary osteoblasts were isolated from *Id4^+/+^* and *Id4^−/−^* neonatal calvarial bone as described previously [35]. Osteogenic differentiation was induced by changing the medium every three days to culture medium supplemented with 100 ng/ml of bone morphogenetic protein 4 (BMP4) (R&D Systems, Mineapolis, MN) and adipogenic differentiation was induced by changing the medium to differentiation medium supplemented with 10% fetal bovine serum (FBS), 0.5 mM 3-isobutyl-1-methlxanthine, 0.25 µM dexamethasone, and insulin-transferrin-selenium-X supplement containing 5 µg/ml of insulin (Invitrogen, Carlsbad, CA) and 1 µM rosiglitazone. After 48 hr, the differentiation medium was replaced with culture medium supplemented with 10% FBS.

### Id4 knockdown by siRNA

siRNA sequences targeting the mouse Id4 transcript were purchased from Ambion (for adipocyte) and Invitrogen (for osteoblast). AllStar Negative Control siRNA (QIAGEN, cat. No., 1027281) and Negative Universal Control Med#2 (Invitrogen, cat. No., 12935-112) was used as a negative control in adipocyte and osteoblast differentiation, respectively. The sequences of siRNA used for Id4 knockdown were as follows; sense, CCUUUGUAUUUGACGUGUAtt; antisense, UACACGUCAAAUACAAAGGtt (Ambion, cat No. AM16704, ID. 159536); for adipocyte differentiation; sense, UUAAUUUCUGCUCUGGCCCUCCCUU; antisense, AAGGGAGGGCCAGAGCAGAAAUUAA (Invitrogen, stealth_455, cat No. 10620312); for osteoblast differentiation. For siRNA transfection, a complex of Lipofectamine 2000 (Invitrogen) and 20 nM siRNA was prepared according to the manufacturer's instruction and directly mixed with cells in the culture plates. The medium was replaced at 4–6 hr after transfection with fresh differentiation medium (adipogenic induction) or 10% FBS supplemented with 100 ng/ml of BMP4 (osteogenic induction).

### Lipid assay and Alkaline phosphatase (ALP) staining

Lipid accumulation in adipocytes was detected by using Oil Red O staining as described previously [36]. The triglyceride content in adipocytes was determined as follows. ST2 cells were washed with cold phosphate-buffered saline (PBS) and total lipids were extracted with chloroform/methanol (2∶1, v/v). The lower organic phase was dried, and the lipids were dissolved in 2-propanol. The triglyceride content was measured using Triglyceride E-test (Wako, Japan) according to the manufacturer's instructions. Protein concentrations were determined with Quick Start Bradford Dye Reagent (BIO RAD, 500-0205), using bovine gamma globulin as standard. ALP staining and measurements were performed as described previously [34].

### Isolation of total RNA and quantitative real-time PCR (qRT–PCR)

Total RNA was isolated from liver, brain, kidney, thymus, brown adipose tissue (BAT), heart, white adipose tissue (WAT), adrenal gland, cortical bone, calvaria, bone marrow and posterior limb of E18.5 mouse embryo using TRIzol reagent (Invitrogen) and RNeasy columns (QIAGEN, Hilden, Germany) according to the manufacturer's instructions. Bone marrow was obtained by slicing of the ends of femur and tibia bones and then flushing it out with PBS using a syringe. Yield and quality of RNA were determined with a NanoDrop spectrometer (NanoDrop Technology, San Diego, CA) and a BioAnalyzer (Agilent Technologies, Santa Clara, CA). Gene expression levels were measured by qRT-PCR as described previously [34]. The sequences of forward and reverse primers used for each gene amplification were as follows; *gapdh*, GAPDH_768Fw_qPCR 5′-TGGAGAAACCTGCCAAGTATG-3′, GAPDH_889Rv_qPCR 5′-GGAGACAACCTGGTCCTCAG-3′; *Id4*, Mm_Id4_532Fw 5′-AGGGTGACAGCATTCTCTGC-3′, Mm_Id4_658Rv 5′-CCGGTGGCTTGTTTCTCTTA-3′; *Pparγ2*, PPARg2Fw 5′-ATGGGTGAAACTCTGGGAGA-3′, PPARg2-1Rv 5′-GAGCTGATTCCGAAGTTGGT-3′; *Bglap1*, OC(PM15547142)Fw 5′-CTCTGTCTCTC TGACCTCACAG-3′, OC(PM15547142)Rv 5′-GGAGCTGCTGTGACATCCATAC-3′; *Adipoq*, Mm_AdipoQ_298Fw 5′-GATGGCACTCCTGGAGAGAA-3′, Mm_AdipoQ_443Rv 5′-GCTTCTCCAGGCTCTCCTTT-3′.

### Expression microarray experiments

RNA was extracted from differentiating ST2 osteoblasts and adipocytes after induction of differentiation, using PureLink miRNA Isolation Kit (Invitrogen) according to the manufacture's instructions for total RNA purification. Total RNA derived from ST2 at 0 hr was used as control. Biotin-labeled cRNA was synthesized as recommended by Affymetrix guidelines. Labeled samples were hybridized to the Affymetrix GeneChip Mouse Genome 420 2.0 arrays according to the manufacturer's protocol. Scanning and intensity data analysis was preformed as described elsewhere [37].

### Expression profiling analyses in ST2 osteoblast and adipocyte differentiation

Collected microarray expression data was background-subtracted, and normalized using the robust multi-array analysis method [37]. Differentially expressed genes were determined by selecting expression subgroups that contained the probe sets of up/down-regulated genes. Genes were considered up-regulated (down-regulated) if their log_2_ intensity ratio was greater (less) than 1 (−1), or greater (less) than the mean plus (minus) 3-times standard deviation. All gene and probe set annotations were derived from Ensembl release 52. Genes with transcription-related Gene Ontology (GO) annotations (Table 3) were considered as transcription factor-coding genes. Genes with InterPro accession IPR001092 were annotated as bHLH transcription factor-coding genes. Spotfire DecisionSite version 8.1.1 (TIBCO Software, Inc.) was used for hierarchical clustering and generating the gene expression heat maps.

**Table 3:** Gene ontology IDs and terms (GO release 2009-01-25) associated with 1,270 transcription factors of Table 1.

GO ID	GO Term
GO:0000156	two-component response regulator activity
GO:0003700	transcription factor activity
GO:0003701	RNA polymerase I transcription factor activity
GO:0003702	RNA polymerase II transcription factor activity
GO:0003704	specific RNA polymerase II transcription factor activity
GO:0003705	RNA polymerase II transcription factor activity, enhancer binding
GO:0003706	ligand-regulated transcription factor activity
GO:0003709	RNA polymerase III transcription factor activity
GO:0003711	transcription elongation regulator activity
GO:0003712	transcription cofactor activity
GO:0003713	transcription coactivator activity
GO:0003714	transcription corepressor activity
GO:0003715	transcription termination factor activity
GO:0003716	RNA polymerase I transcription termination factor activity
GO:0003717	RNA polymerase II transcription termination factor activity
GO:0003718	RNA polymerase III transcription termination factor activity
GO:0008140	cAMP response element binding protein binding
GO:0008148	negative transcription elongation factor activity
GO:0008159	positive transcription elongation factor activity
GO:0016251	general RNA polymerase II transcription factor activity
GO:0016252	nonspecific RNA polymerase II transcription factor activity
GO:0016455	RNA polymerase II transcription mediator activity
GO:0016563	transcription activator activity
GO:0016564	transcription repressor activity
GO:0016565	general transcriptional repressor activity
GO:0016566	specific transcriptional repressor activity
GO:0016943	RNA polymerase I transcription elongation factor activity
GO:0016944	RNA polymerase II transcription elongation factor activity
GO:0016945	RNA polymerase III transcription elongation factor activity
GO:0016986	transcription initiation factor activity
GO:0016987	sigma factor activity
GO:0016988	transcription initiation factor antagonist activity
GO:0016989	sigma factor antagonist activity
GO:0017163	negative regulator of basal transcription activity
GO:0030374	ligand-dependent nuclear receptor transcription coactivator activity
GO:0030375	thyroid hormone receptor coactivator activity
GO:0030401	transcription antiterminator activity
GO:0030528	transcription regulator activity
GO:0042156	zinc-mediated transcriptional activator activity

### Luciferase reporter assays

*Bglap1* promoter regions were cloned by PCR from the mouse genome. 6×E-box sequence (CGCGTCCACGTGGGGCCACGTGGGGCCACGTGGGGCCACGTGGG GCCACGTGGGGCCACGTGGGGA) and cloned fragments were ligated to the firefly luciferase gene (derived from pGL4.10, Promega). CV1 cells were co-transfected with the firefly luciferase reporter vectors, expression vectors and the internal control Renilla luciferase vector (pGL4.74, Promega) using Lipofectamine 2000 (Invitrogen). Luciferase activities were measured with Wallac 1420 Multilabel counter (PerkinElmer Life and Analytical Sciences, Turku, Finland). These experiments were performed in triplicate.

### Co-immunoprecipitation and GST–pull down assay

Cos7 cells were transfected with FLAG-Id4 expression vector with either HA tagged expression vector (Hes1, Hey1, Hey2, Bhlhe40). The extracts from the transfected cells were incubated with Protein G sepharose beads and precipitated with either anti-FLAG antibody (SIGMA) or normal mouse IgG (Santa cruz) overnight at 4C°. After washing with PBS, bound proteins were separated by SDS-PAGE followed by Western blot analysis with anti-HA antibody, anti-FLAG antibody. GST-Id4 expression vector, Flag-Hey2 expression vector and Flag-empty vector were expressed in *E.coli*. The crude extract of GST-Id4 or GST expressed in *E.coli* was incubated with glutatihione sepharose beads for 4 hr at 4C°. The precipitate was incubated with the extract of Flag-Hey2 overnight at 4C°. After washing, the bound proteins were separated by SDS-PAGE followed by Western blot analysis with anti-Flag antibody.

### Chromatin Immunoprecipitation (ChIP)–qPCR analysis

ChIP was performed as described previously [38]. ST2 cells were cultured for 4 days with or without 100 ng/ml BMP4, respectively. The antibodies used for ChIP were anti-Hey2 (Protein Tech Group, cat No. 10597-1-AP), anti-Hes1 (Santa Cruz, H-140) and normal rabbit IgG (Santa Cruz, sc-2027) as negative control. Forward and reverse qPCR primer sequences contained OSE2 of the *Bglap1* promoter (Bglap2_ChIP_L789, 5′-GATTGTGGCCTCTCGTC-3′;Bglap2_ChIP_R8, 5′- ATCGGCTACTCTGTGCTCT-3′).

### Animals

*Id4^−/−^* mice previously generated by Bedford et al. (2005) were kindly supplied by Dr. Kondo who received them originally from Dr. Sablitzky (University of Nottingham). All mice used in this study were maintained and handled according to the protocols approved by the Animal Research Committee of Saitama Medical University.

### Bone morphometric measurements

To assess the static and dynamic parameters of bone histomorphometry, 3 weeks old female mice were labeled with intraperitoneal injections of 20 mg/kg of tetracycline hydrochloride (Sigma) at 4 days before sacrifice. Two days before sacrifice, the mice were injected with 10 mg/kg of calcein (Dojindo Co., Kumamoto, Japan). Tibiae and lumbar vertebrae were removed from each mouse, and fixated with 70% ethanol. The bones were trimmed to remove the muscle, stained with Villanueva bone stain for 5 days, dehydrated in graded concentrations of ethanol, and embedded in methyl-methacrylate (Wako Chemicals, Kanagawa, Japan) without decalcification. Sagittal plane sections (5 µm thick) of the lumbar vertebrae were cut using a Microtome (Leica, Germany). Bone morphometric analyses were performed using a semi-automatic image analyzing system software (System Supply, Nagano, Japan) and Optiphot fluorescent microscope (Nikon, Tokyo, Japan).

## Supporting Information

Figure S1 Heat-map of osteoblast differentiation phase-specific up-regulated transcription factor genes. Transcription factors of the bHLH family are indicated by purple bars. The column ‘Cluster groups’ shows the gene symbols of up-regulated transcription factors in time-sequential (chronological) cluster order. The gene symbols of the up-regulated transcription factors are listed in the same order as they appear on the image. Phase 1 (1hr; 46 transcription factors): Foxc2, Nfatc1, Hoxb2, Fkhl18, Med14, Id4, Msx2, Id2, Ankrd1, Hes1, Bhlhe40, Foxn1, Tle4, Hmga2, Dlx3, Gata2, Dlx2, Nr4a3, Axud1, Eaf1, Hipk2, AC153948.5, Zfx, Cebpb, Grhl1, Smad7, Rel, Maff, Mafk, Klf16, Id1, Nfil3, Fosl1, Klf5, Npas4, Lmcd1, Atf3, Fosb, Mef2c, Erf, Mef2a, Atp6v0a1, Srf, Tgif1, Nr4a1 and Junb; phase 2 (6–24hr; 29 transcription factors); Hey1, Atoh8, Helb, Hdac9, Atf6, Gcom1, Hoxa1, Prrx1, Tgfb1, Creb3l1, Rbm9, Elk3, C1d, Dlx1, Pde8a, Hey2, Ets2, Dtna, Smad6, Foxo1, Cux1, Gatad2b, Irf5, Rfc1, Sp7, Arnt2, Aebp1, Hoxc13 and Aff3; phase 3 (30–48hr; 4 transcription factors): Lef1, Fabp4, Stat5a, Prrx2; phase 4 (4–6d; 7 transcription factors): Hod, Maf, Foxf2, Sox9, Stat1, Foxd1 and Neo1; phase 5 (8–14d; 24 transcription factors): Stat2, Esr1, Klf9, Klf13, AC162313.5, Cml3, Pou6f1, Pura, Nfat5, Zfp521, Trib3, Ddit3, 2210012G02Rik, Clock, Irf7, Nr3c1, Irf9, Zhx2, Sqstm1, Znfx1, Atf5, Hoxa9, Nr1d2 and Nfe2l1.
(1.10 MB TIF)

Figure S2 *Id4* expression pattern in ST2 osteoblast (A) and adipocyte (B) differentiation. Relative expression levels of *Id4* mRNA were measured by qRT-PCR.
(0.16 MB TIF)

Figure S3 4-week-old *Id4* ^−/−^ mice show decrease of growth plate in tibia. (A,B) Villanueva staining of growth plate of *Id4* ^+/+^ (A) and *Id4* ^−/−^ (B) mice tibia. The original magnifications and scale bars of the images are ×64 and 500 µm. (C) Growth plate width in tibia. (D) Longitudinal Growth Rate (Lo. G. R) in tibia. All data were subjected to Student's t-tests. ***p<0.005 versus control. Each error bar represents the mean±SE of *Id4* ^+/+^ (n = 6) and *Id4* ^−/−^ (n = 6), respectively.
(2.67 MB TIF)

Figure S4 Id4 associates with Hey2. (A) Isolation of bHLH transcription factors binding with Id4 was performed by immunoprecipitation (IP). IP was carried out using anti-FLAG antibody and Western blot analysis (WB) was performed using anti-HA antibody. Arrowheads indicate that Hey2 binds with Id4. (B) Direct interaction of recombinant glutathione S-transferase-tagged Id4 (GST-Id4) and recombinant FLAG-tagged Hey2 (FLAG-Hey2) was confirmed *in vitro*. The GST-pull down assay performed using glutathione sepharose beads bound to recombinant GST-Id4. Recombinant FLAG-Hey2 was detected with anti-FLAG antibody.
(0.22 MB TIF)

Figure S5 Expression level of osteoblast marker *in vivo*. (A) Ratio of relative *Bglap1* mRNA expression level between osteoblast-induced POB and non-induced POB from *Id4* ^+/+^ and *Id4* ^−/−^ neonatal calvarial bone. *Id4* ^+/+^ (n = 37), *Id4* ^−/−^ (n = 21). qRT-PCR data were subjected to Student's t-tests. **p<0.01 versus control. (B) Relative expression levels of *Id4* and *Bglap1* mRNA in *Id4* ^+/+^ and *Id4* ^−/−^ mouse embryo (E18.5) posterior limb were measured by qRT-PCR. *Id4* ^+/+^ (n = 1), *Id4* ^−/−^ (n = 4). Each error bar represents the mean±SE of triplicates.
(0.18 MB TIF)

Table S1 List of transcription factors expressed in induced ST2 cells differentiating into osteoblasts or adipocytes. Bhlh members are indicated using bold type. Arrows indicate up- (↑), down-regulated (↓), or up- and down-regulated (↑↓) transcription factors. No change in expression levels is symbolized by the almost equal sign (≈).
(0.10 MB XLS)
