# Supplementary figures and images for: Id4, a New Candidate Gene for Senile Osteoporosis, Acts as a Molecular Switch Promoting Osteoblast Differentiation
Source: PLoS Genet. 2010 Jul 8;6(7):e1001019. doi: 10.1371/journal.pgen.1001019 (PMC2900302; doi:10.1371/journal.pgen.1001019)

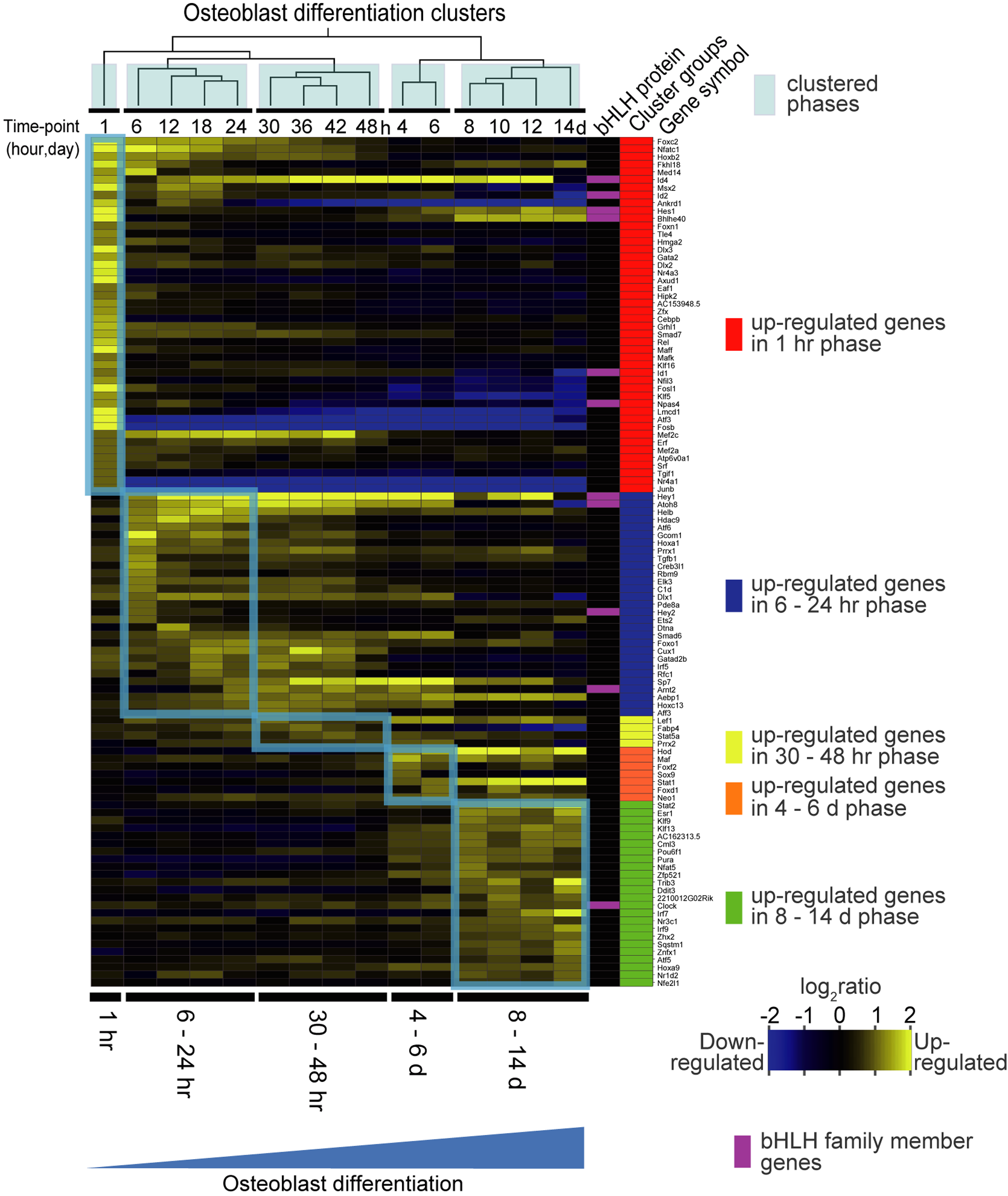

Supplement: Figure S1 — Heat-map of osteoblast differentiation phase-specific up-regulated transcription factor genes. Transcription factors of the bHLH family are indicated by purple bars. The column ‘Cluster groups’ shows the gene symbols of up-regulated transcription factors in time-sequential (chronological) cluster order. The gene symbols of the up-regulated transcription factors are listed in the same order as they appear on the image. Phase 1 (1hr; 46 transcription factors): Foxc2, Nfatc1, Hoxb2, Fkhl18, Med14, Id4, Msx2, Id2, Ankrd1, Hes1, Bhlhe40, Foxn1, Tle4, Hmga2, Dlx3, Gata2, Dlx2, Nr4a3, Axud1, Eaf1, Hipk2, AC153948.5, Zfx, Cebpb, Grhl1, Smad7, Rel, Maff, Mafk, Klf16, Id1, Nfil3, Fosl1, Klf5, Npas4, Lmcd1, Atf3, Fosb, Mef2c, Erf, Mef2a, Atp6v0a1, Srf, Tgif1, Nr4a1 and Junb; phase 2 (6–24hr; 29 transcription factors); Hey1, Atoh8, Helb, Hdac9, Atf6, Gcom1, Hoxa1, Prrx1, Tgfb1, Creb3l1, Rbm9, Elk3, C1d, Dlx1, Pde8a, Hey2, Ets2, Dtna, Smad6, Foxo1, Cux1, Gatad2b, Irf5, Rfc1, Sp7, Arnt2, Aebp1, Hoxc13 and Aff3; phase 3 (30–48hr; 4 transcription factors): Lef1, Fabp4, Stat5a, Prrx2; phase 4 (4–6d; 7 transcription factors): Hod, Maf, Foxf2, Sox9, Stat1, Foxd1 and Neo1; phase 5 (8–14d; 24 transcription factors): Stat2, Esr1, Klf9, Klf13, AC162313.5, Cml3, Pou6f1, Pura, Nfat5, Zfp521, Trib3, Ddit3, 2210012G02Rik, Clock, Irf7, Nr3c1, Irf9, Zhx2, Sqstm1, Znfx1, Atf5, Hoxa9, Nr1d2 and Nfe2l1. (1.10 MB TIF) [file pgen.1001019.s001.tif]

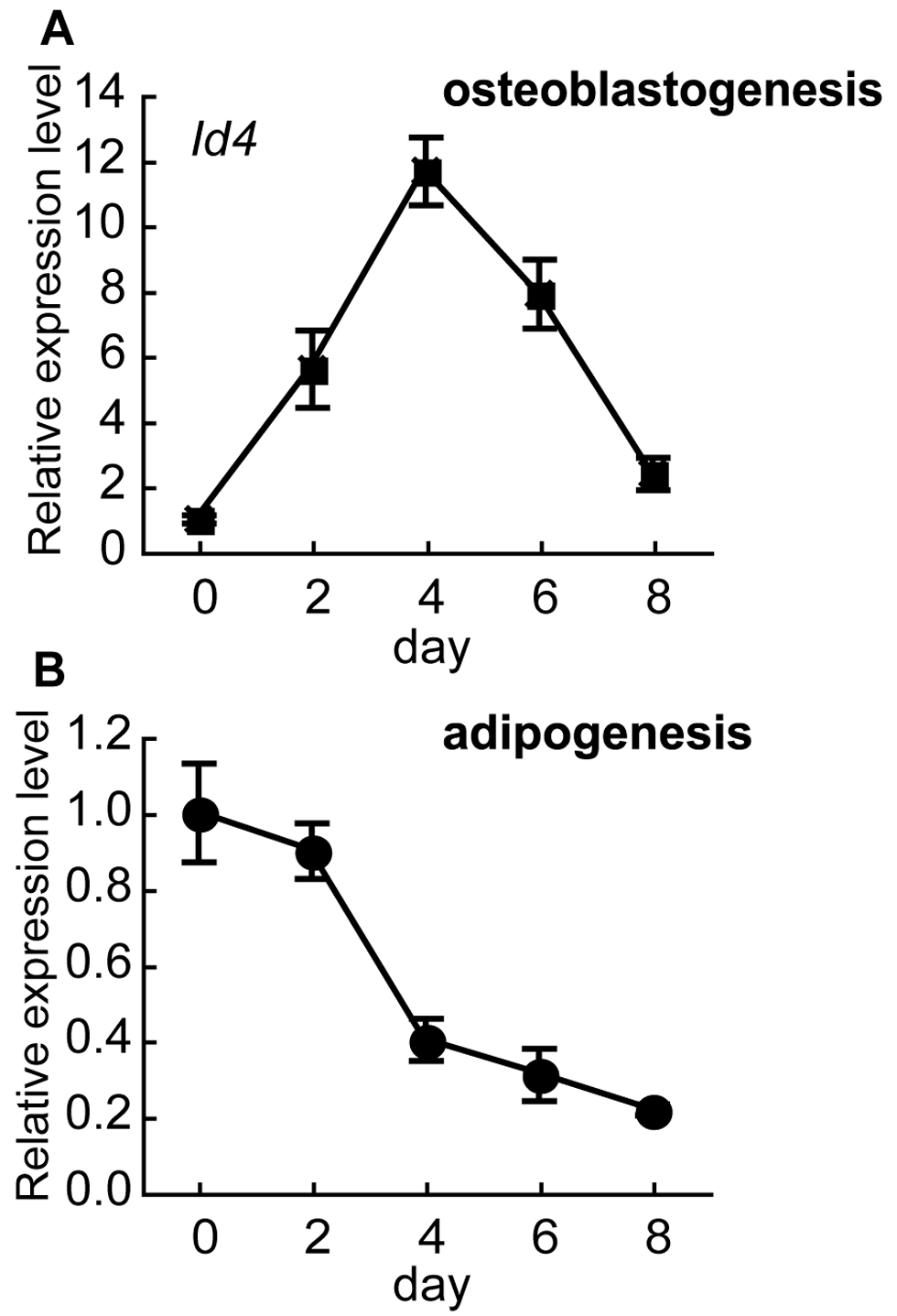

Supplement: Figure S2 — Id4 expression pattern in ST2 osteoblast (A) and adipocyte (B) differentiation. Relative expression levels of Id4 mRNA were measured by qRT-PCR. (0.16 MB TIF) [file pgen.1001019.s002.tif]

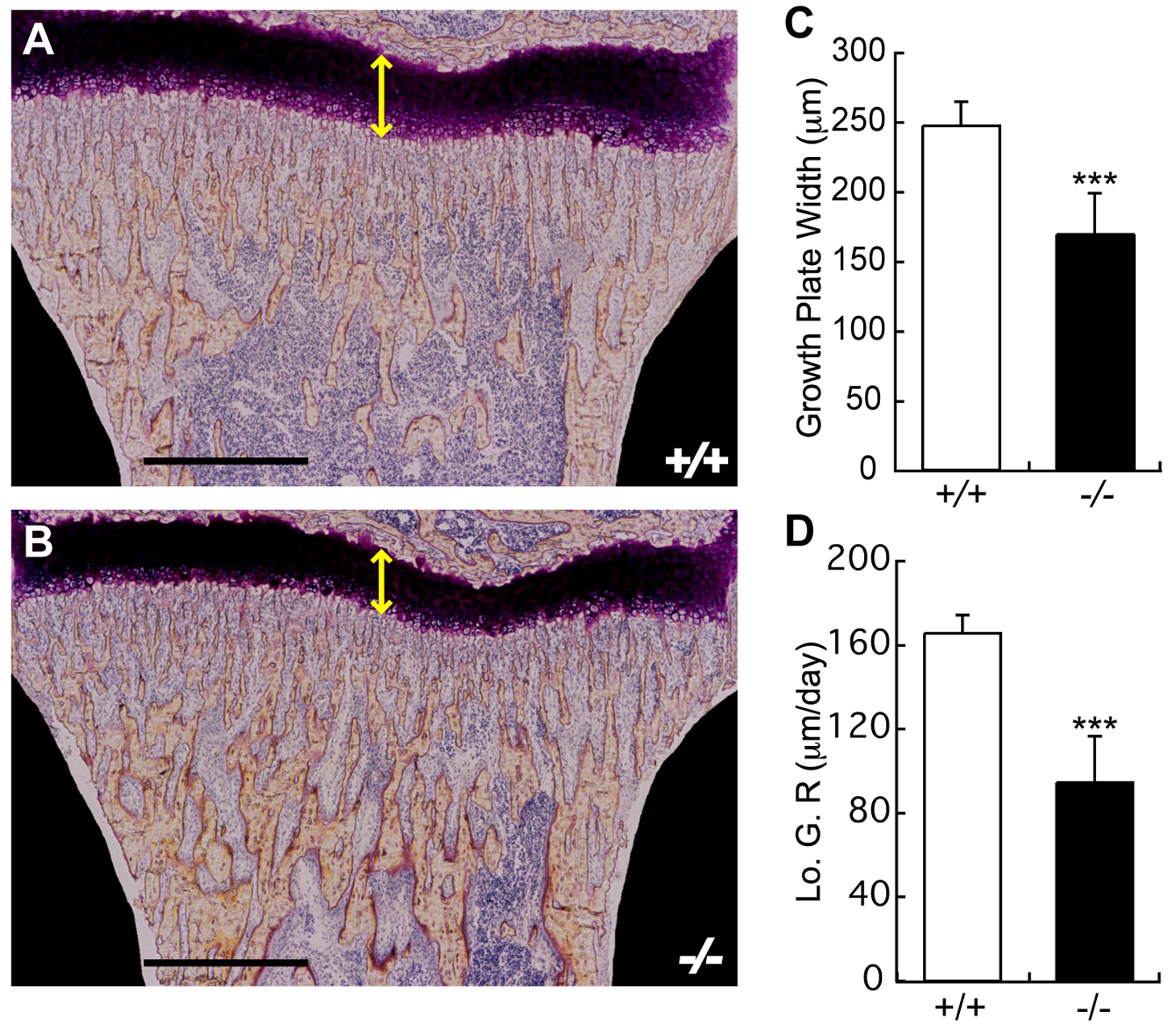

Supplement: Figure S3 — 4-week-old Id4 −/− mice show decrease of growth plate in tibia. (A,B) Villanueva staining of growth plate of Id4 +/+ (A) and Id4 −/− (B) mice tibia. The original magnifications and scale bars of the images are ×64 and 500 µm. (C) Growth plate width in tibia. (D) Longitudinal Growth Rate (Lo. G. R) in tibia. All data were subjected to Student's t-tests. ***p<0.005 versus control. Each error bar represents the mean±SE of Id4 +/+ (n = 6) and Id4 −/− (n = 6), respectively. (2.67 MB TIF) [file pgen.1001019.s003.tif]

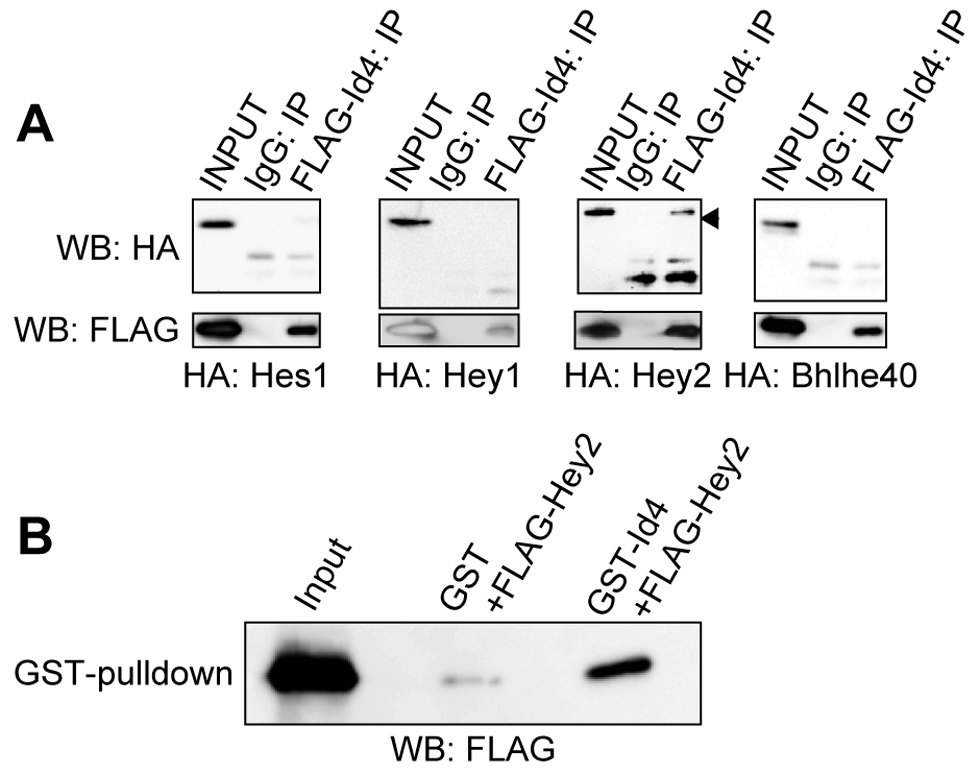

Supplement: Figure S4 — Id4 associates with Hey2. (A) Isolation of bHLH transcription factors binding with Id4 was performed by immunoprecipitation (IP). IP was carried out using anti-FLAG antibody and Western blot analysis (WB) was performed using anti-HA antibody. Arrowheads indicate that Hey2 binds with Id4. (B) Direct interaction of recombinant glutathione S-transferase-tagged Id4 (GST-Id4) and recombinant FLAG-tagged Hey2 (FLAG-Hey2) was confirmed in vitro. The GST-pull down assay performed using glutathione sepharose beads bound to recombinant GST-Id4. Recombinant FLAG-Hey2 was detected with anti-FLAG antibody. (0.22 MB TIF) [file pgen.1001019.s004.tif]

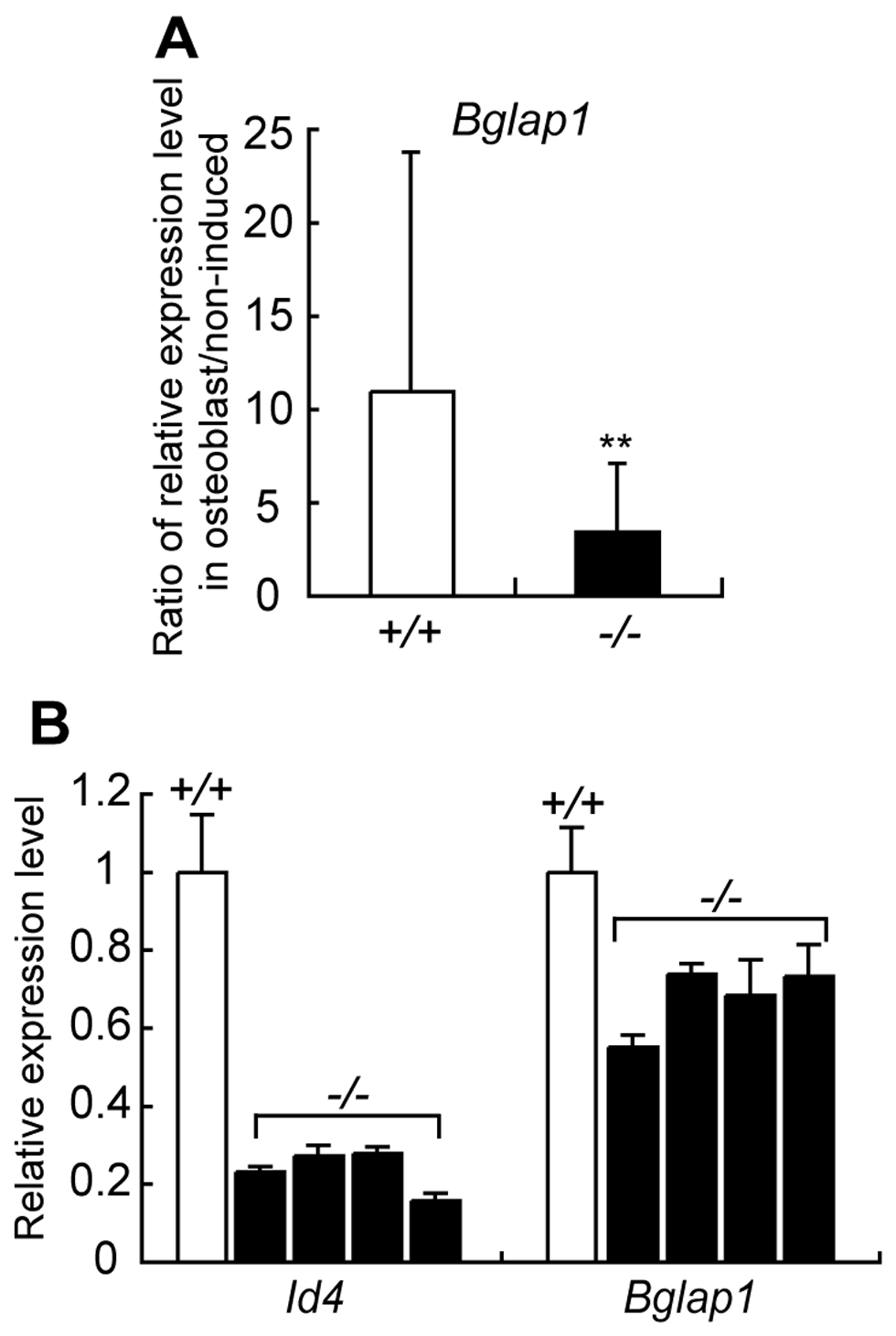

Supplement: Figure S5 — Expression level of osteoblast marker in vivo. (A) Ratio of relative Bglap1 mRNA expression level between osteoblast-induced POB and non-induced POB from Id4 +/+ and Id4 −/− neonatal calvarial bone. Id4 +/+ (n = 37), Id4 −/− (n = 21). qRT-PCR data were subjected to Student's t-tests. **p<0.01 versus control. (B) Relative expression levels of Id4 and Bglap1 mRNA in Id4 +/+ and Id4 −/− mouse embryo (E18.5) posterior limb were measured by qRT-PCR. Id4 +/+ (n = 1), Id4 −/− (n = 4). Each error bar represents the mean±SE of triplicates. (0.18 MB TIF) [file pgen.1001019.s005.tif]
